# The atraumatic restorative treatment (ART) strategy in Mexico: two-years follow up of ART sealants and restorations

**DOI:** 10.1186/1472-6831-13-42

**Published:** 2013-09-08

**Authors:** Elisa Luengas-Quintero, Jo E Frencken, Jorge Alejandro Muñúzuri-Hernández, Jan Mulder

**Affiliations:** 1 Department of Oral Health of the Ministry of Health, Mexico, DF CP 11800, Mexico; 2 Department of Global Oral Health, College of Dental Sciences, Radboud University Nijmegen Medical Centre, P.O Box 9101, Nijmegen 6500 HB, The Netherlands

**Keywords:** Mexico, Health policy, Dental caries, Atraumatic restorative treatment, ART, Glass ionomer, Dental restoration, Pit and fissure sealant

## Abstract

**Background:**

The massive use of preventive measures in Mexico has resulted in a large decline in dental caries over the past two decades. There does however remain a largely unmet need for restorative treatment. This paper describes the steps leading up to the adoption of a strategy, as part of general health policy, to use Atraumatic Restorative Treatment (ART) within the Mexican public health service as a means of addressing this. The objective was to evaluate ART restorations and sealants placed in primary and permanent teeth in schoolchildren from deprived areas over a period of 2 years.

**Methods:**

18 Dentists from 13 municipalities in 6 states with the lowest human development index treated 304, 6- to 13-year-old schoolchildren with ART sealants and ART restorations (single-surfaces) on the school compounds. Ketac Molar Easymix was the filling material used. ART procedures were evaluated according to the ART assessment criteria after 1 and 2 years, by 7 calibrated evaluators. Survival rates were estimated, using the PHREG Model with frailty correction.

**Results:**

The 2-year cumulative survival rates of fully and partially retained ART sealants were 73.1% (primary teeth) and 48.8% (permanent teeth). The dentine carious lesion failure rates of ART sealants in primary and permanent teeth over the 2-year period were 0% and 2.5%, respectively. The 2-year cumulative survival rates of single-surface ART restorations in primary and permanent teeth were 74% and 80.9%, respectively. Secondary carious lesion development occurred in 6 restored primary teeth (2.1%) and in one restored permanent tooth (1.3%). All restorations placed in primary teeth in one state survived, whilst those in one of the 5 remaining states failed statistically significantly more than those in the other 4.

**Conclusions:**

The ART procedures were of substantial quality and had prevented to a large extent the development of new dentine carious lesions in these children from socio-economically deprived areas.

## Background

In the 1970s and 1980s Mexico introduced a number of preventive measures, including the use of fluoride toothpaste, provision at the schools of education on caries prevention and the national program of salt fluoridation (1991). Between 1989 and 2001, the mean DMFT score among 12-year olds declined from 4.4 to 1.91. Of this, the decayed tooth component was 1.54, the missing teeth component 0.04, and the filled teeth component was 0.34 [1]. The prevalence of dental caries in schoolchildren aged 12 years was 58% [1]. Despite the reduction in the burden of dental caries in this age group, a need for restorative treatment remained, which is largely unmet.

### Adoption of the ART approach in Mexico

In 1998 an Atraumatic Restorative Treatment (ART) training course was conducted in Mexico City. The Chief Dental Officer of the Ministry of Health attended it and realised that ART could be very useful for increasing the accessibility and provision of preventive and restorative oral care for deprived communities in the country. In the following years a National Oral Health Program of Mexico for the years 2001–2006 was defined. Actions included: strengthening the curative care, expanding coverage to marginalized localities with problems of access and promoting alternative curative treatment through countrywide adoption of the Atraumatic Restorative Treatment (ART) approach [1]. This approach covers sealing of caries-prone pits and fissures with an ART sealant and using hand instruments and a high-viscosity glass-ionomer in restoring tooth cavities and adjacent pits and fissures (ART restorations) [2]. As these procedures do not require electricity and piped water, ART could be implemented in public health services in marginalized communities.

The plan covered the introduction of the ART approach in public clinics in 19 states selected for their degree of marginalization and lack of access to care [1]. In 2001 a training manual in Spanish was published for national distribution, in preparation for an international master training course on ART held in 2002, [1]. This course was attended by representatives of the Pan American Health Organization, the United States of America Air Force, Cayetano Heredia University of Peru and Caribbean countries, representatives of the 19 priority states and representatives of the health and academic sector of Mexico.

Subsequently, up to the year 2006, 810 dentists were trained in 27 theoretical and practical training courses. In addition, a video on the clinical procedures involved in ART was developed and is integrated into each ART training course. As a result of these initiatives the number of ART procedures provided has continued to increase from year to year. In 2000, a total of 177.823 ART restorations were reported to have been provided in government clinics, rising to 712.869 in 2006. This represented an increase over this period of 400% [1].

In 2007, the National Development Plan and the National Plan for Health 2007–2012 were introduced [1]. One of the strategies included is that of "100 Municipalities 100 Actions". It applies to municipalities which have the lowest Human Development Index (HDI) in the country. A Specific Action Program for Oral Health 2007–2012 outlines 13 strategic actions for improving oral health in Mexico [1]. Strategic Action Number 9 is extending the coverage of dental care through use of Atraumatic Restorative Treatment in the 100 municipalities mentioned above. To achieve this goal, 19 additional ART courses were provided in 2008–2009 to a further 570 dentists, raising the total of number of dentists specifically trained in ART to 1380.

In parallel with the activities introduced to train existing dentists about the ART approach, efforts have also been made to train dental students, with the aim of improving their attitude towards the ART approach as an alternative comprehensive treatment for carious lesions. This will help the newly trained dental graduates, during their obligatory (six months to one year) work in social service, mainly in municipalities with a low human development index.

### The views of Mexican dentists regarding ART

In 2008 in the states of Chiapas, Michoacan and Sinaloa a survey of 197 dentists was undertaken, in order to learn their views concerning the major problems perceived when implementing the ART approach in their practices. The major problem experienced by 45% of the respondents was found to be the scarcity or unavailability of appropriate dental materials and the lack of suitable instruments; those that were available being of poor quality [1].

This survey also identified that just over 55% of the dentists surveyed strictly followed the correct ART approach of using only hand instruments, while the remaining dentists used a high- or low-speed drill, either alone or as a complement to the use of hand instruments, when preparing a cavity for an “ART” restoration. The use of rotary instruments does not feature as part of the ART approach [3], so the number of ART restorations reported from 2000–2006, given above, might be an overestimation. The problem has since been corrected. Between 2007 and 2010, the average number of ART restorations provided was 262.192 per year [4].

### Evaluation of the ART procedures

The Specific Action Program for Oral Health, 2007–2012 points to the need for surveillance of the oral health program for planning and decision making. While this is achieved through the systematized monitoring mechanism that has been instrumental in following, for example, the number of ART procedures provided per year, no information was available regarding the quality of these procedures over time.

In 2008 the National Oral Health Program in Mexico had garnered some six years of experience in using Atraumatic Restorative Treatment as one of its oral health strategies. It is considered to be an important alternative approach in the management of dental caries in marginalized areas of Mexico with problems of access. Therefore, evaluation of ART restorations and sealants placed in accordance with this National Oral Health Program was considered to be appropriate. Consequently a study was designed with the objective of evaluating ART restorations and sealants placed in primary and permanent teeth in schoolchildren over a period of 2 years.

## Methods

### Implementation

A prospective cohort study was conducted in 13 municipalities with the lowest Human Development Index (HDI) in six of the seven states in Mexico with such municipalities. The convenience sample of primary schools was selected. It was based on the track record of the resident dentist(s) trained in ART through the Government training programme and regularly providing oral health activities in primary schools in accordance with the national oral health plan. Dentists were requested to produce some 30 ART procedures in children needing ART sealants or ART restorations over a short period that varied from dentist to dentist, and to record these. Informed consent was obtained from the parents of the participating children and permission for the study was granted by the Ethical Committee of the Department of Training and Research of Ministry of Health of the State Veracruz. It started in 2009.

ART restorations were placed in accordance with standard ART procedures [5]. The ART training course includes a module on caries risk assessment, so sealants were placed in caries-prone pits and fissures in both primary and permanent teeth. Only tooth cavities without pulp involvement were treated. The selection of teeth to be treated was decided by the resident dentist(s) as part of their community dental outreach activities. All treatments were performed within the school facilities, in areas either inside or outside the classrooms. Only single-surface restorations, class I, III and V according to Black's classification, were placed. A single tooth could receive a combination of ART treatments: e.g., an ART sealant on the occlusal surface and a Class V restoration on the buccal surface. Ketac Molar Easymix (3 M ESPE®) high-viscosity glass-ionomer was used for all ART restorations and sealants.

### Evaluation

The evaluations at years 1 and 2 were conducted by the same 7 examiners. They had not been involved in the ART treatments and had been trained over four days in a course delivered by an external international expert. All included children present on the day when the team visited the primary school were evaluated. Evaluations were undertaken using standard ART criteria for assessing the ART restorations and sealants (Table 1). A specially designed form was used for registration and evaluation of the restorations and sealants. Caries was scored at the cavitation level (Table 2). Standard infection control procedures were observed during all examinations.

**Table 1:** ART codes and criteria for assessing restorations and sealants

**ART codes and criteria restoration**	**ART codes and criteria sealant**
0 - Present. Successful, good condition.	0 - Present in all pits and fissures sealed at baseline.
1 - Present. Slight deficiency at cavity margin. (< 0.5 mm in depth).	1 - Any loss of sealant exposing pits and fissures.
2 - Present. Deficiency at cavity margin (≥ 0.5 mm in depth).	
3 - Present. Fracture in the restoration.	
4 - Present. Fracture in the tooth.	
5 - Present. Overextension of approximal margin. (equal to or greater than 0.5 mm).	
6 - Not present. Most or all of the restoration is missing.	6 - Not present.
7 - Not present. Other restorative treatment performed (amalgam, resin, etc.).	7 - Not present. Restorative treatment performed.
8 - Not present. Tooth is not present.	8- Not present. Tooth is not present.
9 - Impossible to diagnose.	9 - Impossible to diagnose.

**Table 2:** Criteria for diagnosing carious lesions in ART studies

0 - No caries	3 - Caries on same tooth surface but not associated with ART restoration or ART sealant extension.
1 - Caries associated with ART restoration	4 - Caries on tooth surface not associated with ART restoration or ART sealant
2 - Caries associated with ART sealant and with loss of sealant extension to ART restoration	9 - Impossible to diagnose.

The examiner reproducibility for all ART procedures was assessed, using Kappa statistics, and was better than 0.82 for inter-examiner reproducibility and better than 0.92 for intra-examiner reproducibility after one-year.

### Statistical analyses

Data were entered into a database and checked for accuracy. The analyses were performed by a biostatistician using SAS version 9.2-software. The dependent variables were the survival rates of ART sealants and restorations. Independent variables were age, gender and region (1–6). The survival rates were obtained after correction for dependency of sealants and restorations within each child. For this purpose, the Jackknife method [6] was applied in calculating standard errors, and the Proportional Hazard Rate Regression Model (PHREG) [7] with frailty correction [8] was used in calculating cumulative survival rates of ART sealants and ART restorations, and analyzing the effect of independent variables on the survival rate over the two-year period. Differences between survival rates were tested, using the Maximum Likelihood test of Wald, sandwich version. Statistical significance was set at α = 0.05.

## Results

### Disposition of subjects

In total 304 children, aged 6–13 years, from 13 primary schools were included in the study. After 1 and 2 years, 245 and 213 children were evaluated, respectively. Information, provided by 18 dentists**,** regarding the background of the participating children and the treatment procedures is presented in Table 3.

**Table 3:** Information on the background of the included children by type of ART procedures in primary and permanent dentitions

	**Age (yrs)**		**Gender**		**FD procedures (%)**			
	**Mean**	**Range**	**Boys (n)**	**Girls (n)**	**N**	**1**	**2**	**3**
ART in primary dentition								
- Sealants	7.0	(5–12)	19	35	99	57	22	14
- Single-surface restorations	6.3	(5–10)	66	84	291	42	35	13
ART in permanent dentition								
- Sealants	7.5	(6–12)	29	57	195	44	20	7
- Single-surface restorations	8.5	(6–13)	24	22	74	54	33	11

*n* number of children, *N* number of procedures, *FD* frequency distribution.

### ART sealants

In primary teeth, all sealants were placed in occlusal surfaces. In permanent teeth 89.2% of sealants were placed in occlusal surfaces, 4.6% in free smooth surfaces and 6.2% of the teeth received sealants in 2 surfaces that included an occlusal surface. The cumulative survival rate of fully and partially retained ART sealants is presented in Table 4. The dentine carious lesion failure rates of ART sealants in primary and permanent teeth over the 2-year period were 0% and 2.5%, respectively. No effects of independent variables tested on ART sealant survival rates in both dentitions over the 2-year period were observed (Table 5).

**Table 4:** Cumulative survival rate (%) and Jackknife Standard Error (SE) of fully and partially retained ART sealants and that of single-surface ART restorations by year of evaluation

	**1 year**			**2 year**		
	**N**	**%**	**SE**	**N**	**%**	**SE**
ART in primary dentition						
- Sealants	82	81.3	4.4	75	73.1	6.2
- Single-surface restorations	239	82.1	2.5	222	74.0	3.3
ART in permanent dentition						
- Sealants	141	72.3	4.3	103	48.8	5.0
- Single-surface restorations	64	86.5	4.2	61	80.9	4.7

**Table 5:** Effects of independent variables on the dependent variables ART sealants and single surface ART restorations over the 2 year period using the Wald (Sandwich) test

**Effects (p-value)**	**Age (yrs)**	**Gender**	**Regions**
ART in primary dentition			
- Sealants	0.11	0.30	0.32
- Single-surface restorations	0.15	0.75	0.009^a^
ART in permanent dentition			
- Sealants	0.98	0.96	0.08
- Single-surface restorations	n/a	0.51	n/a

^a^In one region children had no failed restorations while in one other region, children had significantly more failed restorations than in the remaining regions.

n/a: insufficient numbers in some cells of the matrix.

### ART restorations

The cumulative survival rates of ART restorations in primary and permanent teeth are presented in Table 2. The total numbers of failed ART restorations in primary and permanent teeth were 69 and 13, respectively. Secondary carious lesion development occurred in 6 restored primary teeth (2.1%) and in one restored permanent tooth (1.3%). Other reasons for failure of restorations were related to the mechanical properties of the glass-ionomer used.

In one region all the primary restorations survived, whilst the restorations in one other region failed statistically significantly more than in the four remaining regions (Table 5). No other effect of the independent variables tested on the ART restoration survival rates in both primary and permanent teeth was present.

## Discussion

The introduction of the ART approach into the oral health care system for regions in Mexico with the lowest human development index scores has led to increased access to oral care. The Training the Trainers’ projects, organized by the Dental Department of the Ministry of Health since 2002, resulted in increased placement of ART sealants and ART restorations in children and adults residing in these marginalized regions. The next major step in studying the effectiveness of the introduction of the ART approach in the country dealt with assessment of the quality of the treatments provided. This is covered in the present publication.

Evaluating dental treatments delivered in many remote areas in a large country like Mexico is not easy. For example, all dentists had to be instructed on how to record the baseline data, as this was their first participation in an investigation of this nature. A large number of evaluators had to be recruited. They were inexperienced at the start and very often had to carry out the evaluation alone without a steady recorder, because of logistical problems and financial constraints. Performing a field study under these circumstances is different from participating in a controlled clinical trial in a restricted area. Notwithstanding these drawbacks, consistency in data collection was assured as far as possible, by having the evaluators trained and calibrated in a 4-day educational session prior to the first evaluation year. This was followed by a short rehearsal at the second year of evaluation, led by an international senior epidemiologist. These facts should be taken into account when the survival results of the present study are discussed in a global perspective.

A point of interest was the selection of teeth for review which turned out to contain sealant and single-surface restorations whilst one can assume, with a high level of certainty, that the selected children would also have had teeth with multiple-surface cavities. We can only speculate why this occured. During supervisory visits the first author had seen multiple-surfaces cavities being treated with ART.

Most studies that have evaluated the impact of the ART approach after its introduction into the (oral) health care systems of other countries have merely described the process of how ART was introduced. They have presented numbers of ART treatments over certain periods in countries such as South Africa [9], Tanzania [10], Cambodia [11] and Egypt [12]. Findings regarding the quality of ART sealants and ART restorations are available only from South Africa, where ART was introduced in a mobile setting manned by a limited number (3) of dental practitioners [13]. The one-year survival rate of ART restorations in permanent teeth of primary school children in South Africa was higher (93.6%) than that obtained for comparable restorations in the present study (86.5%). The same restoration assessment criteria were used in both studies. The one year survival rate of ART restorations in permanent teeth in the present study is also lower than the mean one year survival rate (96%) reported in the meta-analysis on ART procedures based on (randomized) controlled clinical trials [14]. A similar pattern is observed for ART restorations in primary teeth.

With respect to the rate of fully and partially retained sealants in permanent teeth, the results from the present study are somewhat lower than those reported in the meta-analysis on ART procedures (82%). The meta-analysis did not include survival rates of ART sealants in primary teeth. The only other study reporting on the survival of fully and partially retained sealants in primary teeth, in which a medium-viscosity glass-ionomer was used, was described in the very first publication on the ART approach [15]. The retention rate was lower in that study (73%) than in the present one (81.3%).

One other study on sealants and restorations placed according to the ART protocol has been conducted in Mexico [16]. The survival rates of ART restorations and of fully and partially retained ART sealants in permanent teeth after two years were lower in the Lopez et al. study [16] than in the present one: 67% vs. 80.9% (ART restorations) and 35% vs. 48.8% (ART sealant retention).

It appears from the foregoing that the survival rates of the ART restorations in both dentitions is somewhat lower than those presented in the meta-analyses [14] but compare well with the only other ART study conducted in Mexico [16]. In the present study, carious lesion development in sealed permanent teeth was very low and equal to the findings reported in the meta-analysis on ART procedures, that in sealed primary teeth was zero, and only 2.1% and 1.3% of ART restorations in primary and permanent teeth, respectively, failed after two years because of secondary caries. This observation is in line with results from other ART restoration studies in permanent teeth, according to a comprehensive review [2]. It shows that the ART approach (sealants and restorations) is able to manage the progression of carious lesions in children effectively, independently of their residential area; whether in a remote marginalized population, as described in the present study, or near a private well-equipped dental surgery [14].

However, it is necessary to note that the survival rates of ART restorations are to a certain extent influenced by the restoration assessment criteria used. These are stricter than the commonly used United States Public Health Services (USPHS) criteria, so the criteria used for assessing ART restorations fail restorations earlier/faster than the USPHS criteria do [14]. The survival of ART restorations in the present study might further be influenced by the handling of the glass-ionomer by the dentists. Some remote areas were situated at high altitude, others at sea level. As a high temperature influences the setting speed of glass-ionomer [17], the geographic location might have interfered with the quality of the mixed glass-ionomer and consequently, with the survival of ART restorations. The dental skills of the participating dentists also need to be taken into account, as operator effects have been reported in ART studies [18]. The fact that all ART restorations in primary teeth in one region survived, while in one of the five remaining regions significantly worse survival rates were observed, shows that the human hand and mind may have influenced the production of quality ART restorations and sealants. Post-graduate education therefore remains essential, although it might be difficult to organise in deprived and remote regions.

The studies that have evaluated the introduction of the ART approach into public health care systems have revealed two major barriers. These are the lack of regular availability of a sufficiently high quantity of high-viscosity glass-ionomer cement and of good quality ART instruments [9-12]. The same reasons were placed high on the perceived list of barriers, by chief dental officers of nine Latin American countries, when introducing the ART approach in the health care systems of their countries [19]. Good dental materials are essential for producing quality dental treatment of any nature. In this context it is worth noting that dentists employed both in public services and in private practice in Egypt were able to perform ART restorations in their private practices but less so in public facilities. This was because of the reduced availability of glass-ionomer cement and proper hand instruments in the public service clinics; materials which they could purchase for use in their own private practice [12].

Ten years ago Mexico introduced an ambitious and forward-thinking policy for improving the oral health of marginalized communities, in part based on a realistic and proven preventive and restorative treatment protocol. The ART procedures were of substantial quality and to a large extent prevented new carious lesion development in the children from deprived areas. The results from evaluating the impact of the ART approach on the oral health of its citizens in terms of numbers and quality of procedures placed, and acceptance by the dental practitioners and the public, indicate that the implementation of this policy should be deemed a success. This does not imply that further improvements are not necessary in this particular field of oral health care. ART is just one component of Mexico’s overall oral health strategy that is firmly based on prevention and on increasing access to oral care for all its citizens. The ART approach should not be restricted to the marginalized regions in the country but should be made available to all other regions as well.

## Conclusions

The dentine carious lesion failure rates of ART sealants in primary and permanent teeth over the 2-year period were very low. The 2-year cumulative survival rates of single-surface ART restorations in primary and permanent teeth were substantial. Secondary carious lesion development in both primary and permanent teeth was low. The ART approach should not be restricted to the marginalized regions in the country. The ART training programme should be extended to all other regions.

## Competing interests

Authors declare that they have no competing interests.

## Authors’ contributions

LEQ conducted the evaluations and ART training and wrote the manuscript. JEF trained the evaluators and master trainers and wrote the study protocol and manuscript. MJAH reviewed the data base, participated in the training of evaluators and reviewed the manuscript. JM analysed the data and wrote the manuscript. All authors read and approved the final manuscript.

## Pre-publication history

The pre-publication history for this paper can be accessed here:

http://www.biomedcentral.com/1472-6831/13/42/prepub

## References

[B1] Hermosillo VH Quintero LE Guerrero ND Suárez DD Hernández MJ Holmgren CJ The implementation and preliminary evaluation of an ART strategy in Mexico: a country example J Appl Oral Sci 2009 17 114 121 10.1590/S1678-77572009000700019 21499665 PMC5467368

[B2] Frencken JE Leal SC de Lima Navarro MF 25 years Atraumatic Restorative Treatment (ART) approach: a contemporary overview Clin Oral Investig 2012 16 1337 1346 10.1007/s00784-012-0783-4 22824915 PMC3443346

[B3] Frencken JE Leal SC The correct use of the ART approach J Appl Oral Sci 2010 18 1 4 10.1590/S1678-77572010000100002 20379674 PMC5349038

[B4] de Salud S Sistema Nacional de información en Salud 2008-2012 2012 Mexico City

[B5] Frencken J Holmgren C Tratamiento Restaurador Atraumático (ART) para caries dentar 1999 Sao Paulo: Santos

[B6] Efron B The jackknife, the bootstrap, and other resampling plans 1982 Philadelphia: SIAM-NSF

[B7] Cox DR Regression models and life tables (with discussion) J R Stat Soc B 1972 34 187 220

[B8] Hougaard P Frailty models for survival data Lifetime Data Anal 1995 1 255 273 10.1007/BF00985760 9385105

[B9] Mickenautsch S Frencken JE van’t Hof MA Factors inhibiting the implementation of the atraumatic restorative treatment approach in public oral health services in Gauteng province, South Africa J Appl Oral Sci 2007 15 1 8 19089091 10.1590/S1678-77572007000100002 PMC4327202

[B10] Kikwilu EN Frencken JE Mulder J Barriers to the adoption of ART as perceived by dental practitioners in government dental clinics J Appl Oral Sci 2009 17 408 413 19936517 10.1590/S1678-77572009000500011 PMC4327665

[B11] Chher T Hak S Courtel F Durward C Improving the provision of the basic package of oral care (BPOC) in Cambodia Int Dent J 2009 59 47 52 19323311

[B12] Farag A Atraumatic Restorative Treatment and oral health in Upper Egypt. PhD thesis 2012 Ipskamp Drukkers BV: Radboud University Nijmegen

[B13] Mickenautsch S Rudolph MJ Ogunbodede EO Frencken JE The impact of the ART approach on the treatment profile in a mobile dental system (MDS) in South Africa Int Dent J 1999 49 132 138 10.1002/j.1875-595X.1999.tb00897.x 10858745

[B14] De Amorim RG Leal SC Frencken JE Survival of ART sealants and ART restorations: a meta-analysis Clin Oral Investig 2012 16 429 441 10.1007/s00784-011-0513-3 21274581 PMC3308010

[B15] Frencken JE Songpaisan Y Phantumvanit P Pilot T Atraumatic restorative treatment (ART) technique: evaluation after one year Int Dent J 1994 44 460 464 7814116

[B16] Lopez N Simpser-Rafalin S Berthold P Atraumatic restorative treatment for prevention and treatment of caries in an underserved community Am J Pub Health 2005 95 1338 1339 10.2105/AJPH.2004.056945 16006415 PMC1449363

[B17] Wilson AD McLean JW Glass-ionomer cement 1988 Chicago: Quintessence Publishing Co 35

[B18] Frencken JE van Amerongen WE Fejerskov O, Kidd E The atraumatic restorative treatment approach Dental Caries. The disease and its clinical management 2008 2 Oxford: Blackwell 439

[B19] Ruiz O Frencken JE ART integration in oral health care systems in latin American countries as perceived by directors of oral health J Appl Oral Sci 2009 17 106 113 10.1590/S1678-77572009000700018 21499664 PMC5467374

